# Medical-Blocks―A Platform for Exploration, Management, Analysis, and Sharing of Data in Biomedical Research: System Development and Integration Results

**DOI:** 10.2196/32287

**Published:** 2022-04-11

**Authors:** Waldo Valenzuela, Fabian Balsiger, Roland Wiest, Olivier Scheidegger

**Affiliations:** 1 Institute for Diagnostic and Interventional Neuroradiology Inselspital, Bern University Hospital University of Bern Bern Switzerland; 2 Support Center for Advanced Neuroimaging, Institute for Diagnostic and Interventional Neuroradiology Inselspital, Bern University Hospital University of Bern Bern Switzerland; 3 Department of Neurology Inselspital, Bern University Hospital University of Bern Bern Switzerland

**Keywords:** biomedical research, data sharing, data handling, data science, platform, software, translational medical research, medical informatics, PACS, DICOM

## Abstract

**Background:**

Biomedical research requires health care institutions to provide sensitive clinical data to leverage data science and artificial intelligence technologies. However, providing researchers access to health care data in a simple and secure manner proves to be challenging for health care institutions.

**Objective:**

This study aims to introduce and describe Medical-Blocks, a platform for exploration, management, analysis, and sharing of data in biomedical research.

**Methods:**

The specification requirements for Medical-Blocks included connection to data sources of health care institutions with an interface for data exploration, management of data in an internal file storage system, data analysis through visualization and classification of data, and data sharing via a file hosting service for collaboration. Medical-Blocks should be simple to use via a web-based user interface and extensible with new functionalities by a modular design via microservices (*blocks*). The scalability of the platform should be ensured through containerization. Security and legal regulations were considered during development.

**Results:**

Medical-Blocks is a web application that runs in the cloud or as a local instance at a health care institution. Local instances of Medical-Blocks access data sources such as electronic health records and picture archiving and communication system at health care institutions. Researchers and clinicians can explore, manage, and analyze the available data through Medical-Blocks. Data analysis involves the classification of data for metadata extraction and the formation of cohorts. In collaborations, metadata (eg, the number of patients per cohort) or the data alone can be shared through Medical-Blocks locally or via a cloud instance with other researchers and clinicians.

**Conclusions:**

Medical-Blocks facilitates biomedical research by providing a centralized platform to interact with medical data in collaborative research projects. Access to and management of medical data are simplified. Data can be swiftly analyzed to form cohorts for research and be shared among researchers. The modularity of Medical-Blocks makes the platform feasible for biomedical research where heterogeneous medical data are required.

## Introduction

Health care institutions are increasingly challenged by the need to balance the increasingly complex clinical pathways and socioeconomic costs. Digital transformation in health care is expected to address this challenge [1]. More accurate and rapid diagnosis, management, and treatment are anticipated through personalized and precision medicine [2,3]. However, combining health care data with biomedical research proves to be difficult and cumbersome for health care institutions, even if the researchers are based at the institutions itself.

Most health care data are available at the level of the health care institutions, often only accessible by clinical personnel and not by biomedical researchers themselves. The availability of data is even more complicated for multicenter research, which is preferable because of the increased sample size, statistical power, and improved generalizability of research [4]. Even if data are available, regulations make data sharing difficult and hinder collaborative research. Although federated learning promises to alleviate the challenge of data sharing, it is a rather new concept that requires expert knowledge, and it is not straightforward to implement. Therefore, the accessibility and sharing of data originating from single or even multiple centers to biomedical research would be advantageous for today’s evidence-based medicine [2,5,6].

Besides the availability of data, the complexity and heterogeneity of data in health care make data-driven biomedical research even more difficult [7-10]. Answering research questions and characterizing diseases often involves diverse and interdisciplinary data [11], ranging from metadata (eg, demographics), clinical information (eg, clinical history and cognitive scores), biological specimens (eg, blood samples), physiological data (eg, electroencephalography), and imaging data (eg, magnetic resonance) to other auxiliary data; that is, multi-omics research. Using such diverse data, a more comprehensive understanding of the diseases and drawing stronger conclusions might be possible [9,12]. However, preparing, handling, and curating heterogeneous data can be tedious and costly [2] before even a single hypothesis can be tested. Knowledge of the available data and means of simple and fast extraction and management of the data from health care institutions are, therefore, key to successful biomedical research.

The development of software platforms facilitating data exploration, management, analysis, and sharing for biomedical research is ongoing, as some previous reviews [9,10,13-15] summarize. Among the numerous existing platforms, those that are most relevant to this work, which are presented on the use case of medical imaging, are summarized hereafter. XNAT (Extensible Neuroimaging Archive Toolkit) [16] is a platform that allows the storage, processing, and sharing of data in biomedical research, with an emphasis on medical images. The virtual skeleton database [17] allows sharing of data in a web-based repository. GIFT-Cloud (Guided Instrumentation for Fetal Therapy and Surgery) [18] is a data sharing and medical image–sharing platform that simplifies the transfer of data from clinics to research. JIP (Joint Imaging Platform) [19] tackles data sharing using a federated approach, which enables the decentralized use of medical images for algorithm development. KETOS [20] is a platform for data analysis, training, and deployment of artificial intelligence (AI) methodologies in health care settings. PRISM (Platform for Imaging in Precision Medicine) [21] handles medical images and associated clinical data, allows the creation of cohorts, and provides image curation functionalities in the setting of the Cancer Imaging Archive. However, most of the available platforms require data to be extracted and curated beforehand and are nonmodularizable; that is, the platforms usually do not provide support if researchers want to use uncommon types of data.

We present Medical-Blocks, a platform that enables exploration, management, analysis, and sharing of data in biomedical research. On the basis of the increasing demand to share and analyze health care data for research, we hypothesize that Medical-Blocks enables swift and secure data exchange. Medical-Blocks can be used as a cloud application or a network of local instances in multi-institutional research, or as a local instance at a single institution, depending on the data sharing and protection regulations. It is adaptable and modularizable to the needs of the particularities of the biomedical research conducted and, hence, the required data.

## Methods

### Overview of Medical-Blocks

Medical-Blocks allows the exploration of data available at clinical systems, management and analysis of these data for research, and sharing of data between institutions for collaborative research. To this end, Medical-Blocks can be connected to data sources of clinical systems (eg, databases such as electronic health records [EHRs] and picture archiving and communication system [PACS]) at health care institutions by blocks. Users can explore the data in the clinical systems through Medical-Blocks, without interfering with the clinical workflow. After identifying data that are suitable for further investigation (eg, within a clinical study), the data can be imported to and managed within Medical-Blocks. Medical-Blocks allows analysis of the data through data visualization, editing, and (automatic) classification by labeling the data such that it becomes research-friendly. By classification of the data, metadata of the data becomes available to the users (eg, number of patients and number of images of a certain type). Therefore, Medical-Blocks allows swift exploration and management of the available data for biomedical research at health care institutions. Furthermore, the metadata or data can be shared through Medical-Blocks from instance to instance or via the cloud in research collaborations.

**Figure 1:**
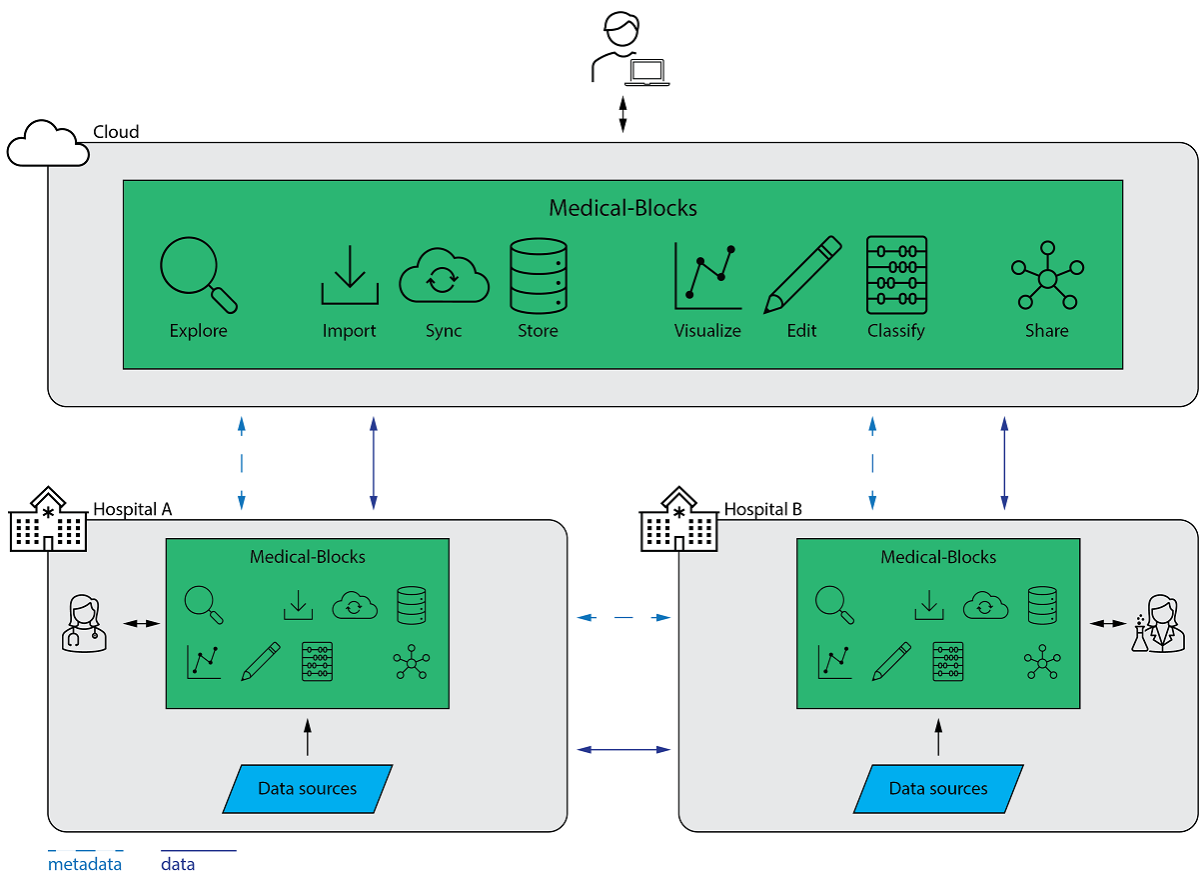
Illustration of the use of Medical-Blocks in a research collaboration between 2 health care institutions via the cloud or direct connection. At each health care institution, a local instance of Medical-Blocks is set up, which accesses the data sources of the health care institution (eg, electronic health records and picture archiving and communications system). Researchers and clinicians can explore, manage, and analyze the available data through Medical-Blocks. For collaboration, metadata (eg, number of patients per cohort) or the data itself can be shared through Medical-Blocks via a cloud instance with other researchers and clinicians. Metadata and data can also be shared directly between local instances of Medical-Blocks from institution to institution.

An exemplary use of Medical-Blocks in a research collaboration between hospitals is illustrated in Figure 1. Medical-Blocks operates both in the cloud and locally at an institution. In both cases, it features the same functionalities. The cloud instance allows users to connect to Medical-Blocks and to perform management and analysis of data from all over the world. Metadata and data can be shared to this cloud instance either from local instances of Medical-Blocks at health care institutions or data can also be imported directly to the cloud, if compliant with the legal regulations (eg, only anonymized data). At health care institutions, Medical-Blocks can be directly connected to the data sources of the clinical systems.

**Figure 2:**
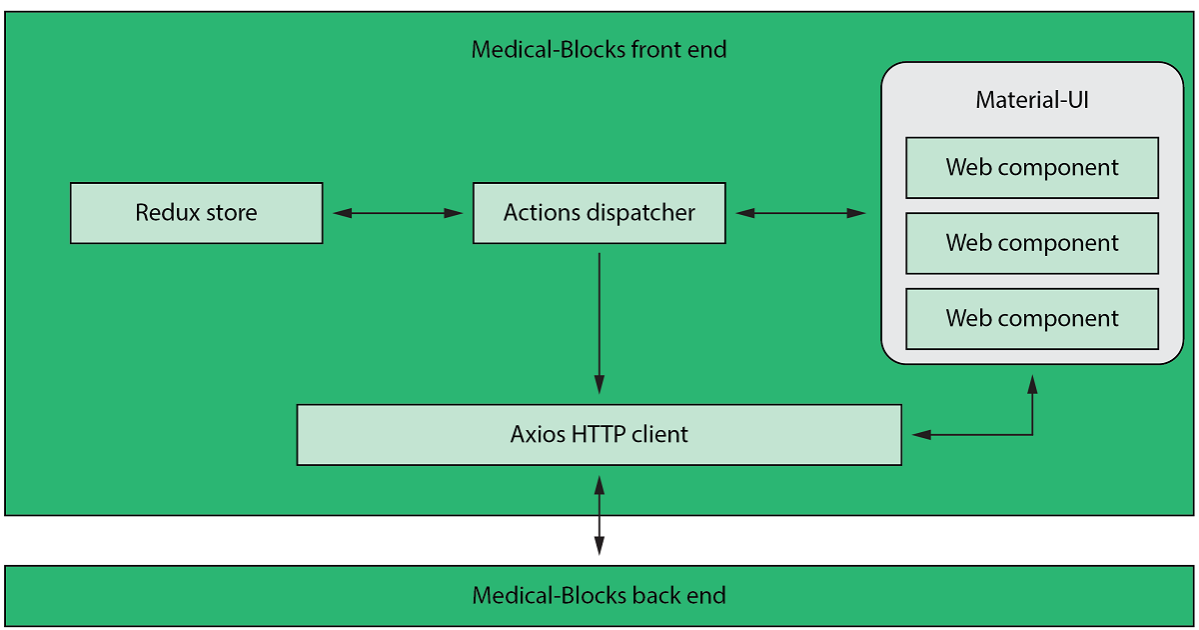
The front end of Medical-Blocks is built using the React library with Material-user interface web components. An Axios HTTP client communicates with the back end of Medical-Blocks. UI: user interface.

Medical-Blocks is implemented as a web application that relies on a client-server model. The implementation of the front end is illustrated in Figure 2. The React library [22] is used to build the user interface (UI) with its web components (tables, combo boxes, etc), which are based on the Material-UI library [23]. The Redux library [24] oversees variables that are used by the web components of the UI and notifies them upon changes in the data. The Axios HTTP client library [25] is used to query, upload, and download data between the web components and the back end’s application programing interfaces (APIs) such as GraphQL and representational state transfer (REST) APIs.

**Figure 3:**
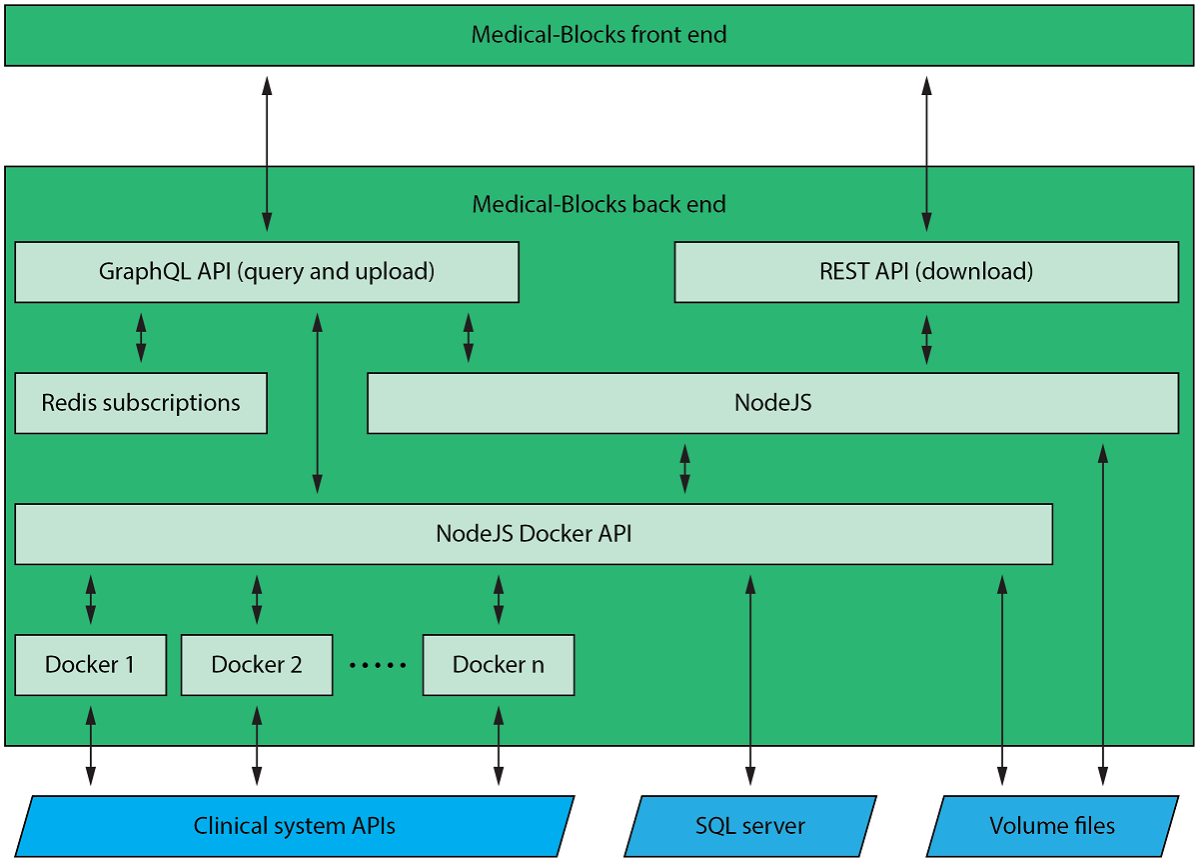
The back end of Medical-Blocks is based on ExpressJS and exposes a GraphQL and download end point. NodeJS is used to communicate with Docker containers—the so-called blocks—that can connect the clinical systems. API: application programming interface; REST: representational state transfer.

The back end is based on the ExpressJS framework [26] that exposes two end points (Figure 3): a GraphQL and REST download end point. The GraphQL [27] end point is implemented using the Apollo server library [28], which handles the query, mutations, and upload events triggered by the clients. Owing to the limitations of the Apollo server library in handling file download events, a download end point was created. The implementation of the download end point was based on the RESTful API. GraphQL ensures communication with the local SQL server through the Sequelize NodeJS library [29]. Files were redirected to an internal files system using NodeJS [30]. To notify clients about events (variables, messages, and new files), we used the subscription system of GraphQL in conjunction with a Redis database [31]. A NodeJS Docker API was implemented to handle the communication to the Docker containers [32]—the so-called blocks. The Docker containers can connect to the APIs of the clinical systems. Details on the technical implementation, such as the PACS and EHR connections, are provided in Multimedia Appendix 1 [27-30,32-38] and referenced accordingly in the subsequent sections.

### Main Features

The main features of Medical-Blocks can be broken down into data exploration, data management, data analysis, and data sharing.

#### Data Exploration

Medical-Blocks can be connected to the data sources of clinical systems, which allows users to explore the data available within an institution. The type of connected data sources, that is, systems and databases, depends on the type of research being conducted. Currently, Medical-Blocks is connected to PACS, EHR, and electroencephalography data sources (section S1 in Multimedia Appendix 1). The connections to the individual systems of the data sources are established through the APIs of these systems by Docker containers (*blocks*) specific to each connected clinical system.

#### Data Management

Medical-Blocks allows the management of data for research, which includes the import of data from data sources to its internal storage system. Data import is possible in three ways: manually by a user over the web UI, semiautomatically using standalone applications (through MB-Connect and MB-Sync described later), or automatically via blocks triggered upon new data being available. For example, the clinical PACS automatically sends a copy of the data (medical image in Digital Imaging and Communications in Medicine [DICOM]) format) to Medical-Blocks. The block processes data into the required format, such as anonymization (eg, removing patient-related information) and conversion to another data format (eg, DICOM to MetaImage conversion). For uncommon data types, the modular architecture of Medical-Blocks allows the integration of new blocks (section S2 in Multimedia Appendix 1).

Medical-Blocks stores data files and information in its own internal system (SQL server and volume files in Figure 3). Storing the data separately allows modification of data such as anonymization and conversion without altering the original data in the institution’s system. The platform provides permanent storage of data prepared for research, which makes the data easily accessible for future research and, therefore, lowers the effort of data collection and preparation. This is in line with the FAIR data principles [39]; that is, findability, accessibility, interoperability, and reusability. Furthermore, the importation to Medical-Blocks lowers the number of requests to the institution’s systems to the minimum, which is only accessed during data exploration and import.

#### Data Analysis

Medical-Blocks allows the analysis of stored data through metadata. Metadata becomes available through analysis blocks that automatically classify and label newly stored data upon import or by manual triggering. Such metadata could be, for instance, the type of disease or the imaging sequence, which is described further in section S3 in Multimedia Appendix 1. As the analysis depends on blocks and the modularity of Medical-Blocks allows the integration of new blocks (section S2 in Multimedia Appendix 1), the type of analysis performed, and therefore the metadata, is user- and project-specific. If an automatic extraction through blocks is not possible, metadata can also be added by the user manually. Data can also be visualized and inspected using the built-in viewers in the platform (eg, image viewer for images).

The metadata provides a research-friendly summary of the available data via a dashboard. This summary might facilitate the creation of potential cohorts for research, which is often a time-consuming process. Therefore, metadata offers the potential to explore the available data in a more research-driven manner. Such exploration is usually not provided by clinical systems, which rarely come with features that facilitate research, as they function at the level of individual patients rather than cohorts.

#### Data Sharing

Data sharing is one of the core features of Medical-Blocks. A built-in file hosting service via the cloud permits sharing of data, similar to well-known file hosting services such as Dropbox. The extent of sharing is freely configurable by providing individual users, groups of users, and even users from other institutions access to the cloud. Therefore, Medical-Blocks meets the requirements of biomedical research, where collaboration is often key to success.

The data can be shared on two levels: (1) sharing of metadata and (2) sharing of full data. The sharing of metadata allows the exchange of summaries of the available data based on the data analysis performed in Medical-Blocks. Therefore, researchers can explore the available data without sharing the actual underlying data. In research involving multiple groups and multicenter research, sharing metadata allows exploring potential collaborations regarding aspects such as data set size and data composition. As only metadata are shared, the potential abuse of data is prevented. As soon as all stakeholders agree, Medical-Blocks then allows researchers to exchange the full data that underlies the metadata.

### Design Principles

The design of Medical-Blocks adheres to five principles: (1) simplicity, (2) flexibility, (3) modularity, (4) scalability, and (5) security.

#### Simplicity and Flexibility

Medical-Blocks is accessible via a web UI, which allows researchers to interact with various data formats available at health care institutions within one interface. Domain knowledge regarding clinical systems and access to software specific to data formats (eg, PACS use and access) is not required for researchers. Furthermore, the web UI makes the platform agnostic to specific hardware and operating systems requirements.

Metadata simplifies the exploration of potential cohorts for research through a dashboard view of the UI. This contrasts with accessing different clinical systems to search for potential cohorts, which can be a tedious process depending on the number of clinical systems involved. Beyond the dashboard, technically versed users can also use the GraphQL playground for exploration (section S4 in Multimedia Appendix 1).

Data sharing and access to data are further facilitated by standalone sync applications that can be installed on PCs, which reduces interactions with the web UI and allows files to be uploaded to Medical-Blocks via the file explorer. Their functionality is very similar to well-known file hosting services; that is, shared data are directly synchronized to the file system and are accessible via the file explorer of the operating system. There are two versions of the sync application: a full version (MB-Sync) and a lightweight version (MB-SyncLight). The lightweight version works only unidirectionally; that is, data are only synced from the platform to the client. This also allows sharing of data with users who are not registered users of Medical-Blocks by providing a token for access. The full version works bidirectionally; that is, data can be synced from Medical-Blocks to the client, and vice versa. The sync applications are available for the operating systems Windows, macOS, and Linux. Details of the technical implementation are provided in section S5 in Multimedia Appendix 1.

Medical-Blocks offers various features that facilitate project management, as synchronizing the communication between multiple researchers and keeping track of the current state of a research project are often cumbersome. This is further complicated if multiple institutions and researchers are involved in multiple projects. Medical-Blocks facilitates project management through a communication, notification, and activity logging system. Users can access the status of a project and review what other users have been doing in the project, if new data are available, among others. Using the communication system, users can communicate with each other and with the teams to which they are assigned.

#### Modularity

Medical-Blocks is modularizable to adequately cope with the complexity of the information technology (IT) ecosystems of modern hospitals, such as multiple vendors, different APIs, and security restrictions. Individual patient data are typically stored in various systems at an institution (eg, clinical, laboratory, and radiology). To obtain an entire view of the electronic medical record of a patient, the data needs to be pooled from these individual systems, which can be a cumbersome process for researchers because of the different interfaces to access the systems. Medical-Blocks simplifies access to data by using blocks tailored to connect to the clinical systems through their APIs. These blocks allow a flexible adaptation of Medical-Blocks to the IT ecosystem of the health care institutions and for different research projects. Depending on the type of research project, a block can be integrated to access data from a previously unconnected clinical system.

#### Scalability

Medical-Blocks is intended for use at various levels of operations. The first level is the use as a cloud instance or local instance at a health care institution without any connection to clinical systems; that is, data are imported manually through the web UI. The next level is the connection to the clinical systems of the health care institution. Further levels are then the connection to other Medical-Blocks; that is, from institution to institution and to the cloud. The connection to the clinical systems is possible in two ways: (1) by directly connecting a Medical-Blocks instance and (2) by using MB-Connect. Connecting Medical-Blocks necessitates a local instance running on a server, which may not always be desired and feasible. Therefore, MB-Connect, a software plug-in, can be used at health care institutions as a bridge to a cloud instance of Medical-Blocks (section S6 in Multimedia Appendix 1). Therefore, the use of Medical-Blocks can be adjusted depending on the requirements of the health care institutions and the size of the research collaboration.

Scalability is directly linked to the available resources Medical-Blocks runs on. To ensure scalability, Medical-Blocks leverages operating system virtualization; that is, the main core of Medical-Blocks is designed as containers that store and run their corresponding functionality. Using Kubernetes (Cloud Native Computing Foundation), the containers can be scaled according to the live demand of resources. Depending on the estimated maximum resource requirements, Medical-Blocks can run on low-cost hardware such as Raspberry Pi (Raspberry Pi Foundation) to enterprise products such as Google Cloud (Google Inc). Hardware can be locally installed, virtualized, and cloud-based. Easy scalability is especially important as big data and data-driven methods are becoming more prevalent in biomedical research [3,5,10], which will result in an increased demand for the storage and management of data. Furthermore, having the possibility of running instances at a smaller scale allows the inclusion of smaller institutions and their data owing to relatively flexible hardware requirements.

#### Security

Security is a key requirement for software that interacts with health care data. The security and privacy of health care data are usually regulated at the national or international level; for example, in the United States through the Health Insurance Portability and Accountability Act of 1996 and in the European Union through the General Data Protection Regulation. Therefore, software interacting with health care data must adhere to the regulations of the countries in which the software is being deployed. In Switzerland, the management of health care data for research requires at least three main security features (Ordinance on Clinical Trials in Human Research 810.305; Article 18): (1) restricted access, (2) user rights, and (3) traceability of operations.

Medical-Blocks provides restricted access, user rights, and traceability of operations performed on data. Restricted access is enforced by a secure log-in to the platform (section S7 in Multimedia Appendix 1). Rights can be assigned at the user level to prevent unwanted import, access, and modifications. All operations (ie, import, access, and modifications) performed on the data by the system and users are logged and saved for a potential audit. Therefore, Medical-Blocks adheres to the common legal and ethical regulations in biomedical research. It must be noted that such features are not necessarily implemented in clinical systems (eg, clinicians often have access to all patients without specific restrictions).

The user management of Medical-Blocks allows to define roles from the level of projects to teams, down to the level of single users. The principal investigator can define the data, teams, and users involved in a project. To simplify user management, teams of users can be formed with team-wide rights, which can be assigned to projects. Rights can also be defined at the user level; for instance, clinicians can access deanonymized data, whereas researchers can only access anonymized data. Generally, data imported into Medical-Blocks gets assigned to the user who performs the import, which is the first measure to prevent abuse of data as it is only accessible by this user. Furthermore, data exploration and import are restricted to specific users to prevent unauthorized access to clinical systems. Users can be restricted to only see metadata instead of the true underlying data. Similar to exploration and import, data sharing is also restricted to specific users.

### Medical-Blocks at the Inselspital

We present Medical-Blocks on the use case of medical imaging and how the platform is currently being used at the Inselspital (University Hospital of Bern, Bern, Switzerland). This use case encompasses mostly research in the field of quantitative medical image analysis, involving the processing of medical images using AI developed to extract quantitative imaging biomarkers for monitoring of treatment response and as an outcome measure. To do so, researchers need to have access to medical images acquired in daily clinical routine to develop and evaluate AI methodologies on real-world data. To date, this process has been tedious because it involves accessing the PACS of the hospital to query and retrieve medial images of potential cases in the DICOM format. Subsequently, researchers had to anonymize and convert the DICOM images to a regulatory-complying and research-friendly format. Furthermore, the medical images had to be linked to complementary (clinical) information such as demographic variables and diagnoses extracted from other clinical systems.

**Figure 4:**
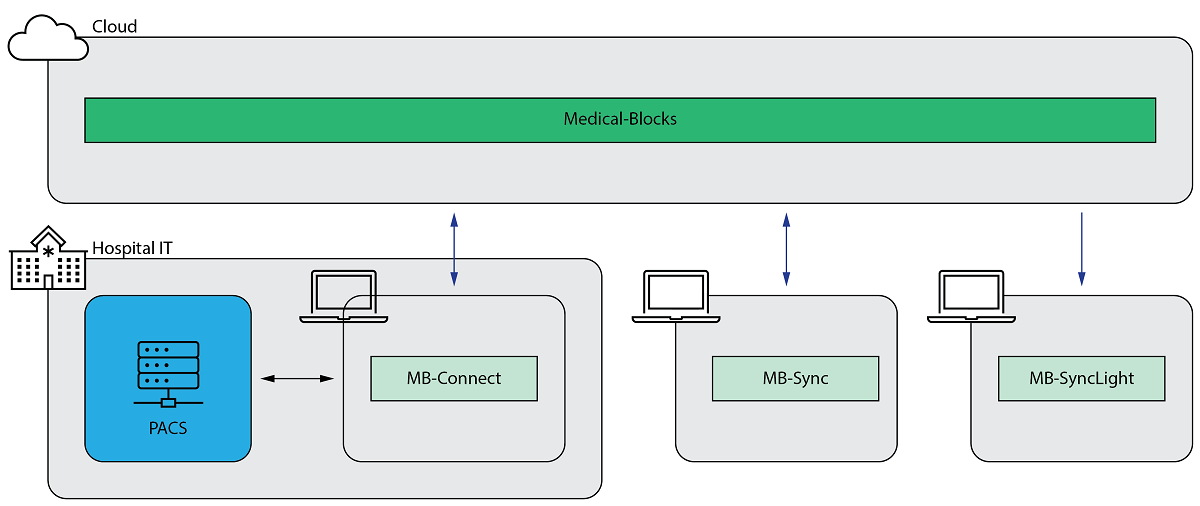
Overview of Medical-Blocks as used at our hospital. Owing to legal regulations, the picture archiving and communications system cannot be directly connected to Medical-Blocks as patient-identifying would be shared over the internet. Therefore, we use the MB-Connect plug-in within an in-house Digital Imaging and Communications in Medicine viewer for uploading anonymized medical images to Medical-Blocks. Users of Medical-Blocks can access the data via the web user interface. Synchronization of data to the user’s file systems is possible by two synchronization applications (MB-Sync and MB-SyncLight). IT: information technology; PACS: picture archiving and communication system.

Medical-Blocks was integrated into the IT imaging ecosystem at our hospital (Figure 4). We opted to use Medical-Blocks as a cloud instance, which does not necessitate the installation of Medical-Blocks at the hospital but, in turn, necessitates that all data contained in Medical-Blocks must be anonymized to comply with the legal regulations of the responsible authorities. Therefore, we use MB-Connect to access the unanonymized data of the PACS, anonymize the data, and send the data to Medical-Blocks in a semiautomatic manner. MB-Connect was integrated into an in-house DICOM viewer as a plug-in (MB-Viewer; section S6 in Multimedia Appendix 1). Upon import, the users of Medical-Blocks can access the data via the web UI from anywhere. Furthermore, the data can be synchronized and shared with any computer by two synchronization applications: MB-Sync and MB-SyncLight.

## Results

We present the results of the development of Medical-Blocks separated into the main features of data exploration, data management, data analysis, and data sharing.

### Data Exploration

**Figure 5:**
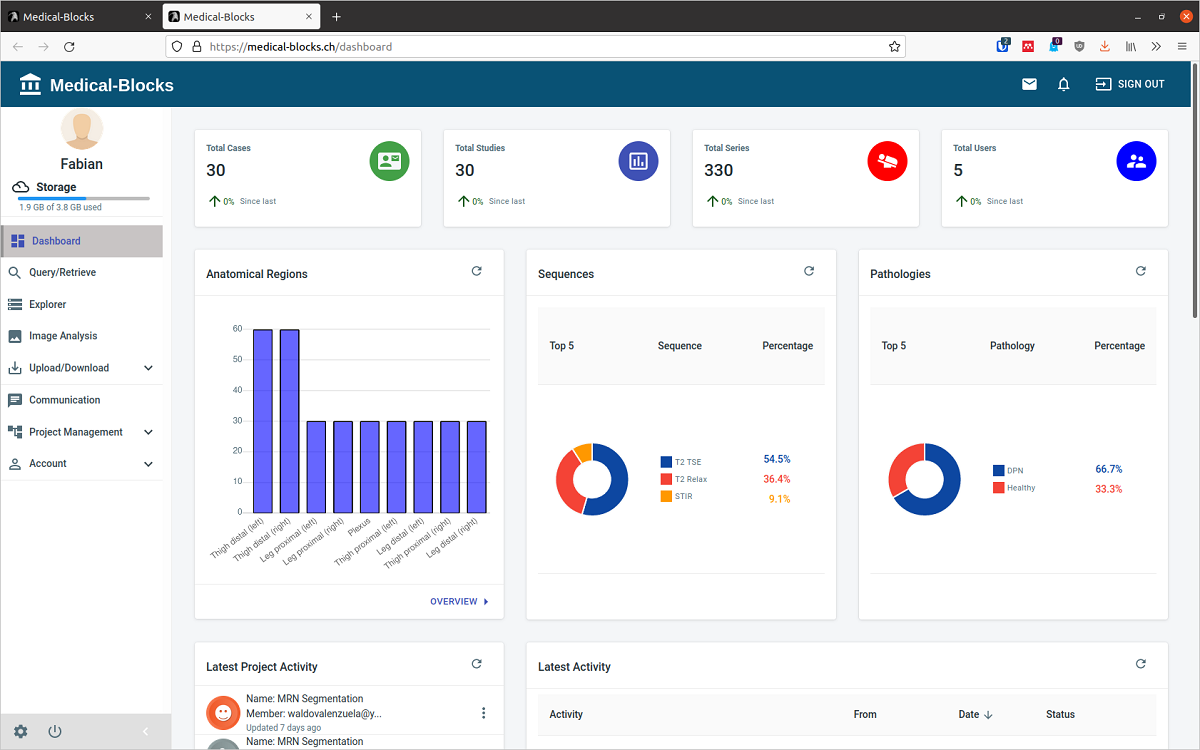
Dashboard of Medical-Blocks shown upon log-in to the platform. The dashboard visualizes the metadata; that is, it provides a concise summary of the available data.

The dashboard with a summary of the metadata is presented to the user upon log-in into Medical-Blocks (Figure 5). The number of cases, studies, and series available become directly visible to the user. It also presents summaries on anatomical regions, sequence, and the type of pathology. Furthermore, the dashboard presents the latest activities within the project to the user. Moreover, only the metadata and activities of the project or projects to which the user has access are shown.

**Figure 6:**
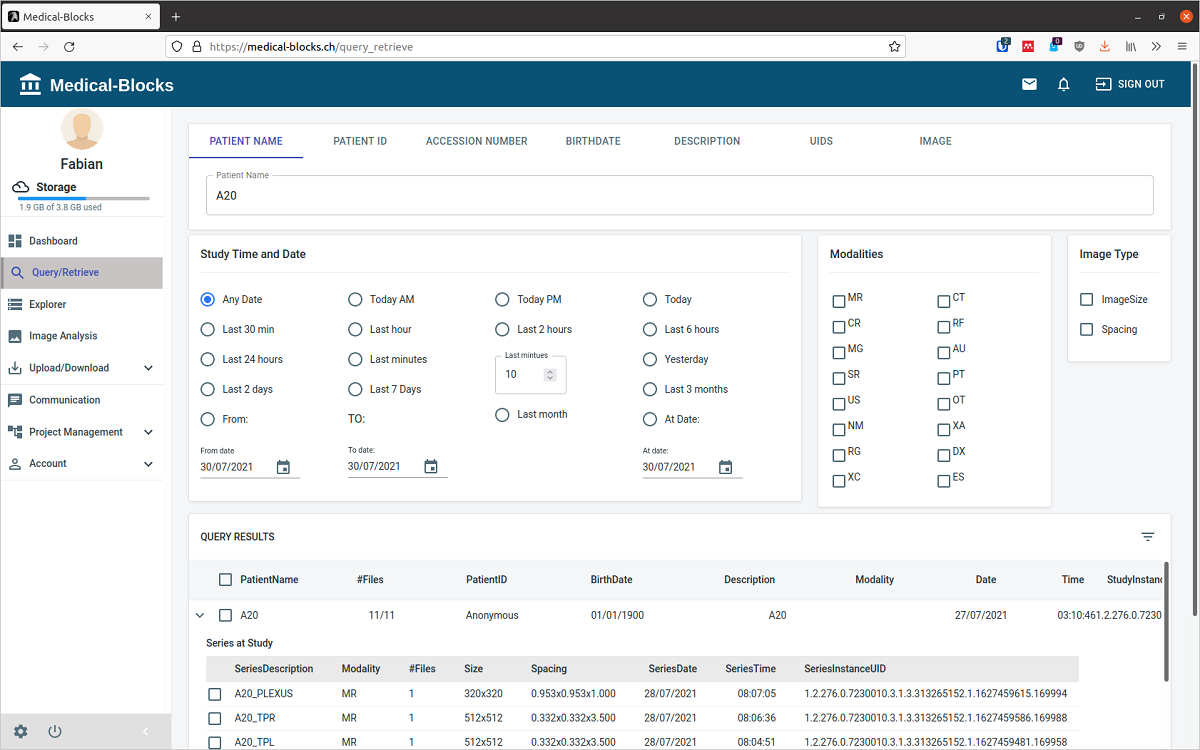
Data exploration through the Query/Retrieve section. Upon entering a patient name, the available data in Medical-Blocks search for matching entries, which are listed in the query results. The query can be refined by restricting it to a certain date or a range of dates, imaging modalities, and image properties.

Exploring available data in the clinical systems at the institution and in Medical-Blocks is possible in the Query/Retrieve section (Figure 6). Querying of data is similar to that in commercial PACS software: querying by patient name, patient ID, accession number, date of birth, study description, unique identifiers, and image properties. The query can be refined by date, image modality, and image properties options. A query will list all results that match the search criteria. If Medical-Blocks is not directly connected to the PACS of the hospital, it only retrieves results from the data contained within the platform. If Medical-Blocks is connected to a PACS, a query lists the results from the PACS that can be explored and imported without the need of having access to the actual clinical systems (the PACS viewer in this case). This feature can be limited to certain users of Medical-Blocks to prevent abuse. Medical-Blocks further ensures that all queries and imports are logged.

### Data Management

**Figure 7:**
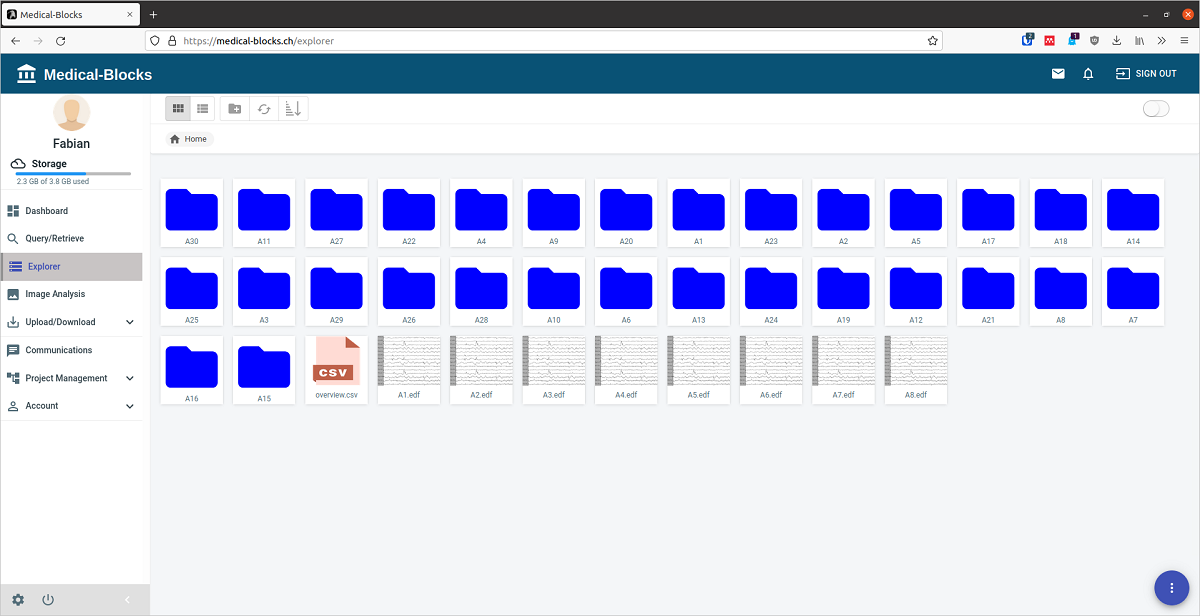
Overview of the available data in Medical-Blocks through the Explorer section. The explorer allows files to be managed in a manner similar to that of file explorers in current operating systems. Here, 30 folders containing image data, 1 CSV file, and 8 electroencephalography files are present.

The Explorer section of Medical-Blocks allows the inspection of available data in the platform (Figure 7). The Explorer section works like explorers known from today’s operating systems. It allows the user to rearrange files into folders, copy files, cut files, paste files, and remove files. The explorer is agnostic to the type of data; that is, electroencephalography or text documents are also displayed in the Explorer section. Furthermore, the explorer has a drop feature that allows users to import a file directly in the Explorer section, facilitating the way of moving files to Medical-Blocks for sharing.

**Figure 8:**
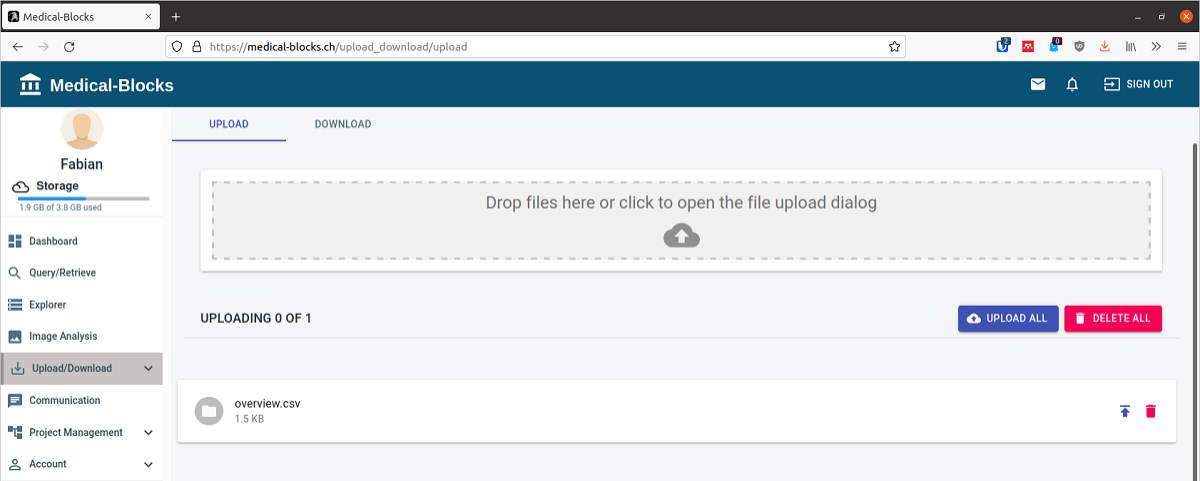
Manual import of data to Medical-Blocks. Files to be imported can be selected using a file system dialogue or by dropping the files to the user interface of Medical-Blocks.

Import of data to Medical-Blocks is possible through the Query/Retrieve section (if Medical-Blocks is connected to a clinical system), MB-Connect, MB-Sync, and manually. The manual Upload/Download section (Figure 8) extends the import capabilities of the explorer to multifile import. The file or files to be imported can be selected from the file system of the computer by a file system dialogue or directly imported by dropping to the Upload/Download section. Once imported, the files become visible in the explorer.

### Data Analysis

**Figure 9:**
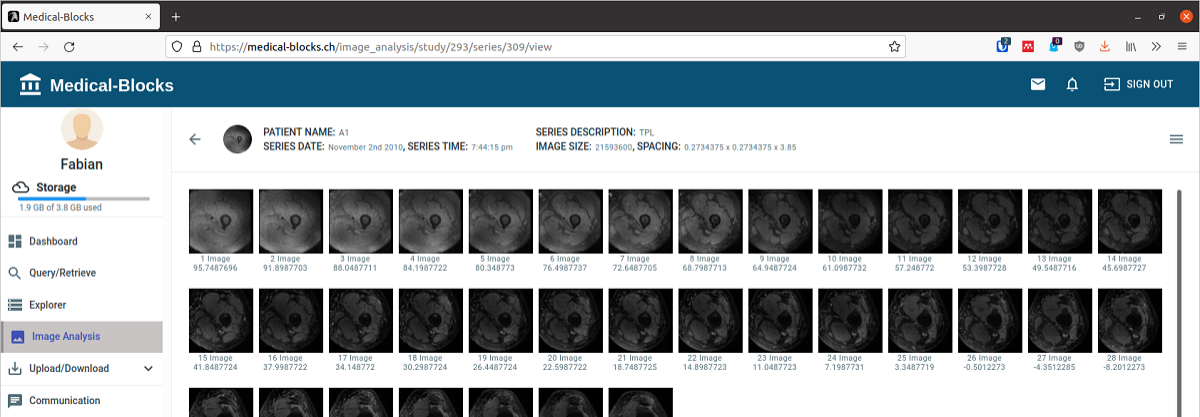
The built-in viewer allows to inspect the different image slices of a medical image within Medical-Blocks.

**Figure 10:**
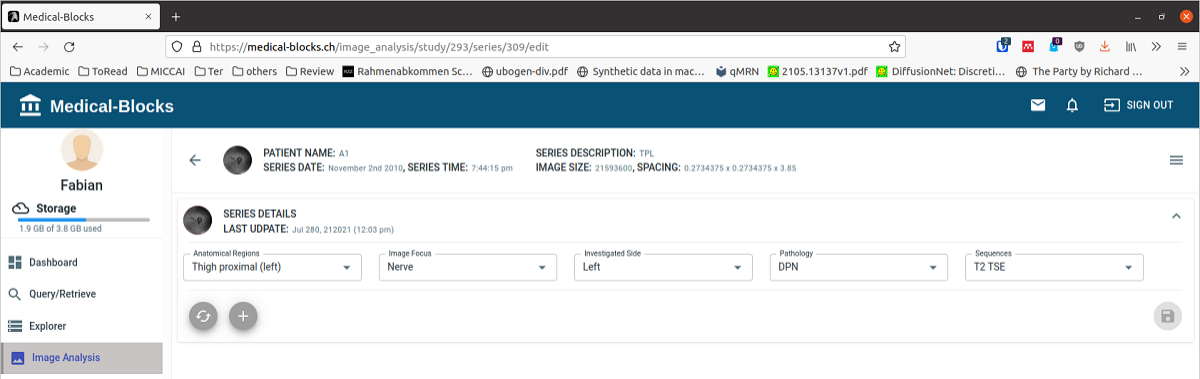
The process of manually classifying data in Medical-Blocks. By selecting a medical image, it can be classified according to anatomical region, image focus, investigated side, pathology, and sequence.

Medical-Blocks presents a summary of the data available in the form of metadata in the dashboard of Medical-Blocks (Figure 6), which allows a high level of automation in the data analysis. Furthermore, the built-in viewer allows, for example, the inspection of medical images directly via the web UI (Figure 9). A section for manual classification appears when selecting a file (Figure 10). This section allows to correct wrong classifications and to add user-defined classifications that are not automatically extracted by the blocks.

### Data Sharing

**Figure 11:**
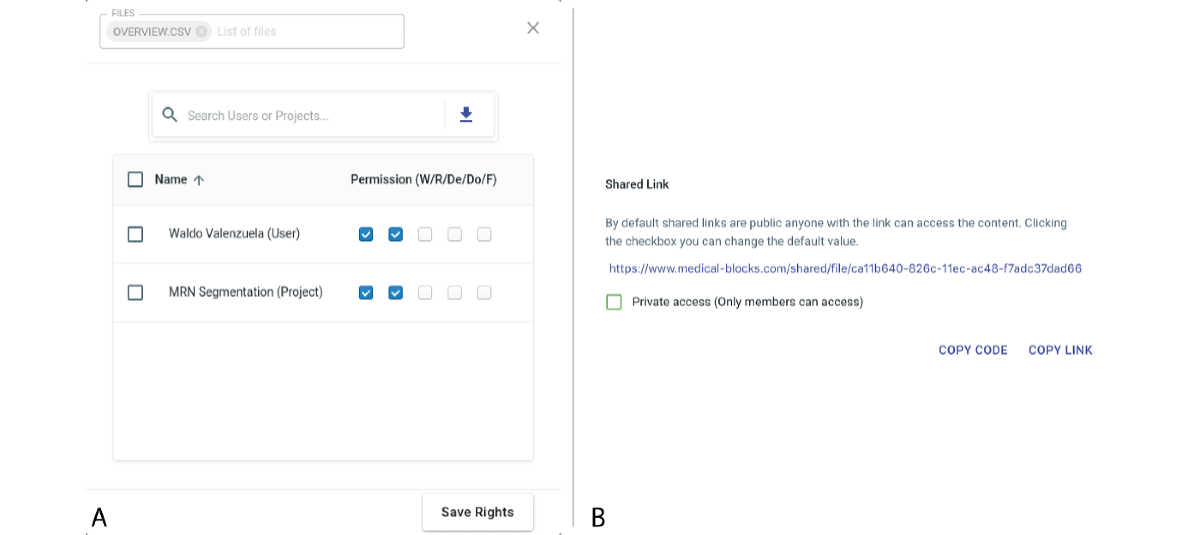
The sharing of data in Medical-Blocks. (A) The owner of the data within the explorer gives read access to the data to another user (Waldo Valenzuela) and a project (MRN Segmentation). The files will now appear in the explorer dialogue of the user Waldo Valenzuela and for all users assigned to the project MRN Segmentation (with appropriate user rights to view data). (B) Share links for direct sharing of data can be automatically generated.

Sharing of data via Medical-Blocks is possible in multiple ways. First, users see the metadata of the available data in Medical-Blocks on a project-level in the dashboard by default (Figure 5). Second, the owner can provide access to the data to other users or projects in a corresponding dialogue of the explorer or by generating a share link, as shown in Figure 11. As soon as access rights are granted, data will appear in the explorer of the other user or users. Third, synchronization applications MB-Sync and MB-SyncLight can be used for sharing.

**Figure 12:**
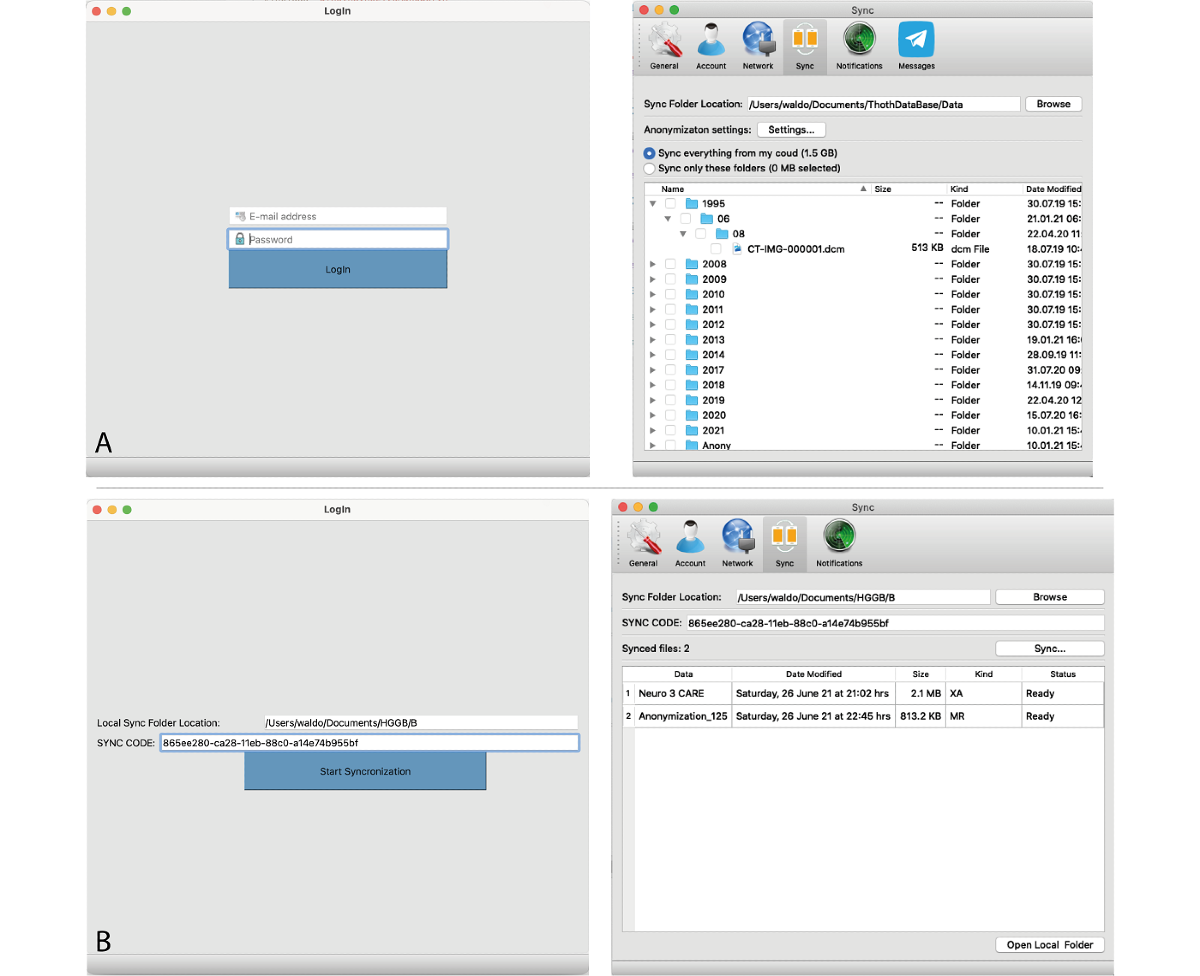
The synchronization applications MB-Sync (A) and MB-SyncLite (B). For MB-Sync, the user uses the log-in credentials of Medical-Blocks and selects which data to sync and to which location. For MB-SyncLite, a person receives a synchronization code (SYNC CODE) that grants access to a specific folder (here HGGB). In both cases, the data are synced to the file system and are accessible via the explorer of the operation system.

Using the synchronization applications, data from Medical-Blocks can be synchronized to any computer’s file system, as shown in Figure 12. Access to data can be granted on a folder level in the explorer; that is, by sharing a link to a user of the synchronization application (Figure 11). Data access can also be granted to people who are not users of Medical-Blocks by generating a SYNC CODE (Figure 12). This code can be used with MB-SyncLite to retrieve data from Medical-Blocks without being a user of the platform.

### MB-Connect

**Figure 13:**
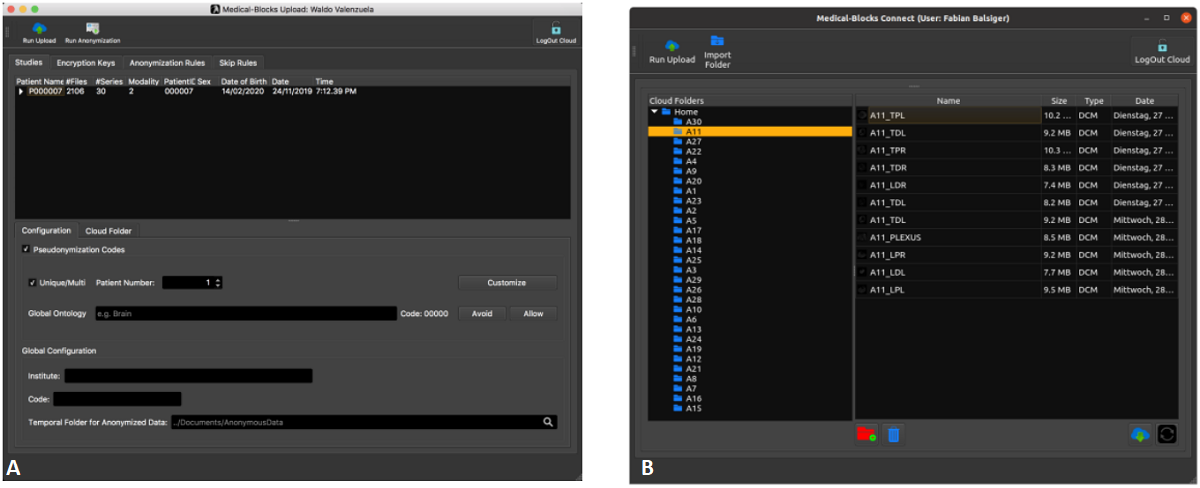
The anonymization (A) and upload (B) dialogues of MB-Connect integrated into our in-house Digital Imaging and Communications in Medicine (DICOM) viewer MB-Viewer. Before the upload of medical images to Medical-Blocks, the DICOM tag fields need to be anonymized by using the dialogue shown (A). The user specifies to which directories on Medical-Blocks the medical images are uploaded to by using the dialogue shown (B).

MB-Connect is used to import data to the cloud instance of Medical-Blocks (Figure 13). MB-Connect was integrated as a plug-in into an in-house DICOM viewer called MB-Viewer (section S6 in Multimedia Appendix 1). By default, the DICOM files to be imported are anonymized using a predefined template (eg, the date of birth is set to January 1, 1900). If required, the user can edit and modify the anonymized information, that is, which DICOM tag fields will be anonymized according to what rules, through the anonymization dialogue (Figure 13A). For the import to Medical-Blocks, the user can select the directories to which the medical image or images will be imported (Figure 13B). By default, the medical images will be uploaded to the user’s home directory, as with the Upload/Download section in the web UI (Figure 8). The directories on Medical-Blocks can also be directly modified within the upload dialogue such as editing the directory name as well as creating and deleting directories. Anonymization is mandatory before the upload of medical images to Medical-Blocks such that no patient-identifying information is being uploaded to the cloud instance of Medical-Blocks.

## Discussion

We conceptualized and devised Medical-Blocks to enhance the exploration, management, analysis, and sharing of data in collaborative biomedical research. The platform can be connected to clinical systems for direct exploration of data for potential research. Data imported into and managed by Medical-Blocks are available to other researchers for further analysis. Visualization and classification of data allow the formation and analysis of potential cohorts for research. As Medical-Blocks can run as a cloud application, sharing of metadata and data with collaborators is easily possible, enabling multicenter research. An ecosystem of complementing software such as MB-Connect and synchronization applications MB-Sync and MB-SyncLight further extend the applicability and usability of Medical-Blocks. Medical-Blocks is accessible for use on the web [40]. New users must register, and access is granted upon reasonable request.

Data analysis and data sharing are two key features of Medical-Blocks. The automatic analysis of data allows the convenient exploration of data to form new cohorts for research through metadata. This metadata allows further exploration of potential collaborations with other researchers by sharing the type and extent of data available without sharing the underlying data. Once cohorts are defined, the underlying data can easily be shared with collaborators. The synchronization applications MB-Sync and MB-SyncLight make sharing and synchronizing data to the file system straightforward.

By connecting the medical systems of a health care institution, medical data become accessible to researchers who usually do not have direct access to such systems. This allows the exploration of available data for potential research without interfering with the clinical workflow. By managing the data with Medical-Blocks, the data are handled in a standardized manner independent of proprietary data formats. Researchers are likely to spend less time on converting and managing data because the platform can automate such processes.

The integration of computational blocks into Medical-Blocks is a feature that is currently lacking. In the use case of medical imaging, computational blocks can, for instance, leverage AI for medical image analysis. Such computational blocks can be used in different ways to classify data for metadata and research purposes. For the classification of metadata, AI can automatically predict the investigated side, which would further automate the data analysis if not simply possible through DICOM tag fields. For research purposes, AI is used for medical image analysis such as segmentation [41,42], brain morphometry [43], and reconstruction [44,45]. By executing such blocks when new data are synchronized from the PACS and when a user imports new files, AI can be tested on real clinical data acquired in everyday clinical practice. Therefore, a novel AI can be deployed in a shadow-mode–like environment for the continuous validation of AI [46].

A major hurdle in developing Medical-Blocks was its integration into the hospital IT infrastructure. Directly connecting Medical-Blocks to the PACS of the hospital underlies legal restrictions related to cloud-based data transfer. Running Medical-Blocks as a local instance and connecting it to the PACS was possible without any problems, as the use of MB-Connect highlights. Nevertheless, to develop and leverage data sharing—a key feature of the platform—we opted to use Medical-Blocks as a cloud instance. We believe that this was the right trade-off; that is, fully leveraging data sharing while restricting the connection to clinical systems. This setting also shows that Medical-Blocks can be used without having a local instance running, but only by using MB-Connect integrated into a DICOM viewer for the data exploration and upload of data from the PACS to a cloud instance of Medical-Blocks. This setting might further make it simple to convince smaller institutions to participate in a multicenter research project, as no local instance of Medical-Blocks needs to be run in the institution’s IT infrastructure.

We will address several shortcomings with the next release of Medical-Blocks. First, we aim to certify the platform such that it can manage unanonymized medical data in the cloud; that is, a certification as a medical device. A direct connection to the clinical systems at our hospital without intermediate software such as MB-Connect might then be possible. Having unanonymized data available for multicenter research might benefit the classification, cohort exploration, and ultimately the conclusions of the research projects. Second, we aim to apply Medical-Blocks beyond the use case of medical images. A first step in this direction was already made by starting a project involving electroencephalography data, but a more diverse set of types of data would be favorable for research involving multiple medical disciplines. Third, the integration of computational blocks involving AI is a key strategy for future releases (section S2 in Multimedia Appendix 1). Researchers should be able to add their AI as blocks to the platform and run these blocks directly on the newly imported data. Such a possibility could hopefully facilitate the application of novel AI in shadow mode before translating it to clinical practice. Finally, we believe that the ongoing and increasing use of Medical-Blocks will likely reveal several aspects we currently do not think about but are key to better user experience and more accurate and faster biomedical research.

In conclusion, we introduced Medical-Blocks that facilitates biomedical research by providing a centralized platform to interact with medical data in collaborative research projects. Medical-Blocks simplifies access to and management of medical data. Data can be analyzed swiftly to form cohorts for research. Finally, data can be shared among researchers. The modularity of Medical-Blocks makes it possibly applicable to various types of biomedical research involving heterogeneous medical data.

## Appendix

Multimedia Appendix 1 Technical implementation details.

## Abbreviations

AI: artificial intelligence
API: application programing interface
DICOM: Digital Imaging and Communications in Medicine
EHR: electronic health record
IT: information technology
PACS: picture archiving and communication system
REST: representational state transfer
UI: user interface
