# Supplementary material for: Medical-Blocks―A Platform for Exploration, Management, Analysis, and Sharing of Data in Biomedical Research: System Development and Integration Results
Source: JMIR Form Res. 2022 Apr 11;6(4):e32287. doi: 10.2196/32287 (PMC9039815; doi:10.2196/32287)
Supplement: Multimedia Appendix 1 [file formative_v6i4e32287_app1.pdf]

Multimedia Appendix for Valenzuela et al. Medical-Blocks—A Platform for Exploration, Management, Analysis, and Sharing of Data in Biomedical Research: System Development and Integration Results

S1 PACS, EHR, and EEG connections

The PACS connection is implemented using two approaches. In both cases, Medical-Blocks has its own DICOM node. In the first approach, it is a simple PACS listener implemented by a Docker version of the DCMQRSCP application provided by the DCMTK library [1]; DCMQRSCP has been adapted to be used in conjunction with SQL databases. In the second approach, it uses a DCM4CHEE PACS internally to handle the data traffic of larger infrastructures (we used the DCM4CHEE Docker [2] for the PACS). The communication between the DCM4CHEE PACS and Medical-Blocks is implemented by listening to the volume files without the need of using DICOMWeb. For both approaches, the DICOM files are handled by a manager, which stores the data on the file system and manages the relational information (e.g., linking to projects, ownership, folder locations) in an SQL database. The storage is either anonymized or not. To query and retrieve data from the UI, we use a modified MOVESCP Docker that triggers the query/retrieve from the clinical PACS. A flowchart of the PACS connection is shown in Figure 1.

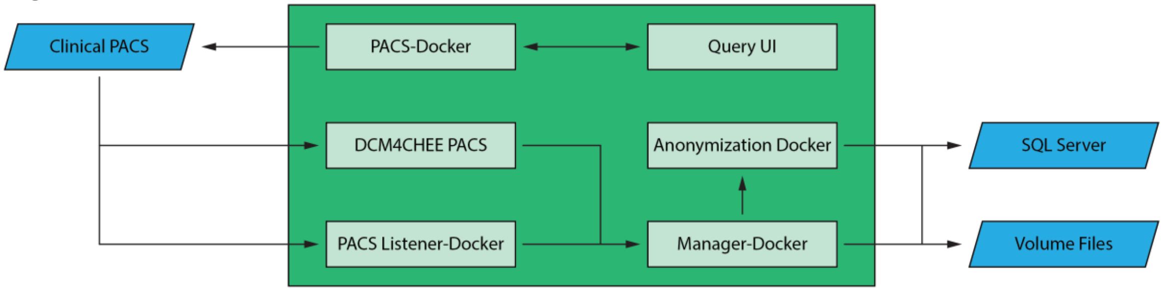

Figure 1. Flowchart of the PACS connection.

The EHR connection is implemented by a direct communication to the SQL database of the clinical EHR system. We use the sequelize library [3] to query and retrieve data from this SQL database, which is stored in the SQL server of Medical-Blocks. The Apollo GraphQL server [4] inside Medical-Blocks handles the interaction with the stored EHR data. The flowchart of the EHR connection is shown in Figure 2.

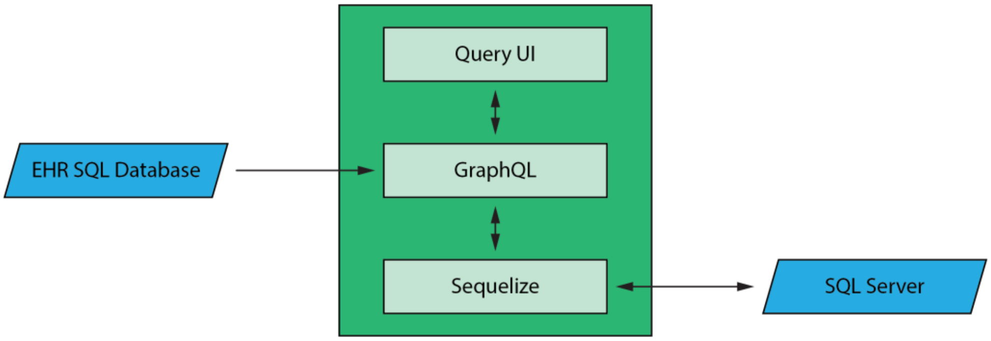

Figure 2. Flowchart of the EHR connection.

The EEG file system of the hospital is connected through MB-Sync. MB-Sync monitors changes in the EEG file system and send a copy of the EEG file in the EDF format [5] to Medical-Blocks, which stores the data inside the file storage. Like the EHR connection, the Apollo GraphQL server handles the interaction with the stored EEG data. The flowchart of the EEG connection is shown in Figure 3.

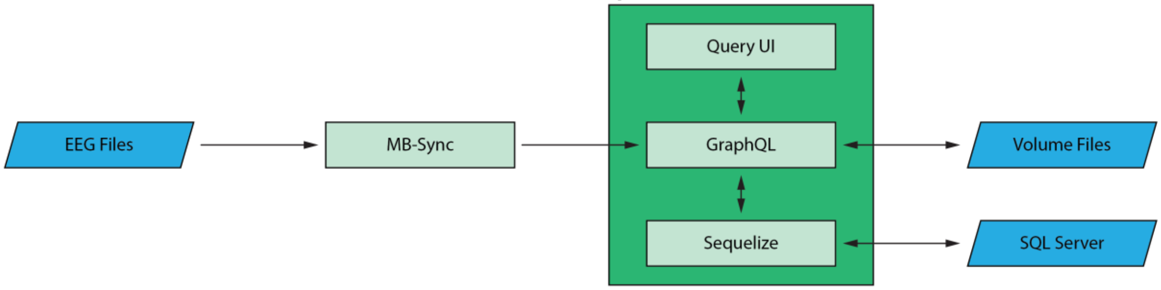

Figure 3. Flowchart of the EEG connection.

S2 Docker system

Medical-Blocks is implemented using different Docker containers [6] for back-end, front-end, databases, and blocks for data analysis. Based on the containerized approach of Medical-Blocks, the deployment is possible in different ways (Kubernetes, Docker Swarm, Docker Compose). The current deployment bases on Docker Compose through the YAML file with the Portainer interface [7] due to hardware restrictions.

Custom-made Docker containers – the so-called blocks – can be integrated into Medical-Blocks by the user manually. We implemented a Node.js [8] Docker API (cf. Figure 3 of the back-end in the main manuscript) to manage these Docker containers (create, start, and delete of containers). The web UI allows managing these Docker containers as shown in Figure 4. New blocks can also be added through the web UI, which allows for easy integration of new user-specific blocks like an AI-based algorithm. In the UI, the user can select if a block will be executed automatically as soon as a new file arrives that belongs to the user of the block (see also A5 on access rights) or executed manually by the user when selecting a file. Access to the block can also be given beyond a single user to the level of a project and team. The output of the blocks will be stored in the file system of Medical-Blocks and be accessible through the explorer.

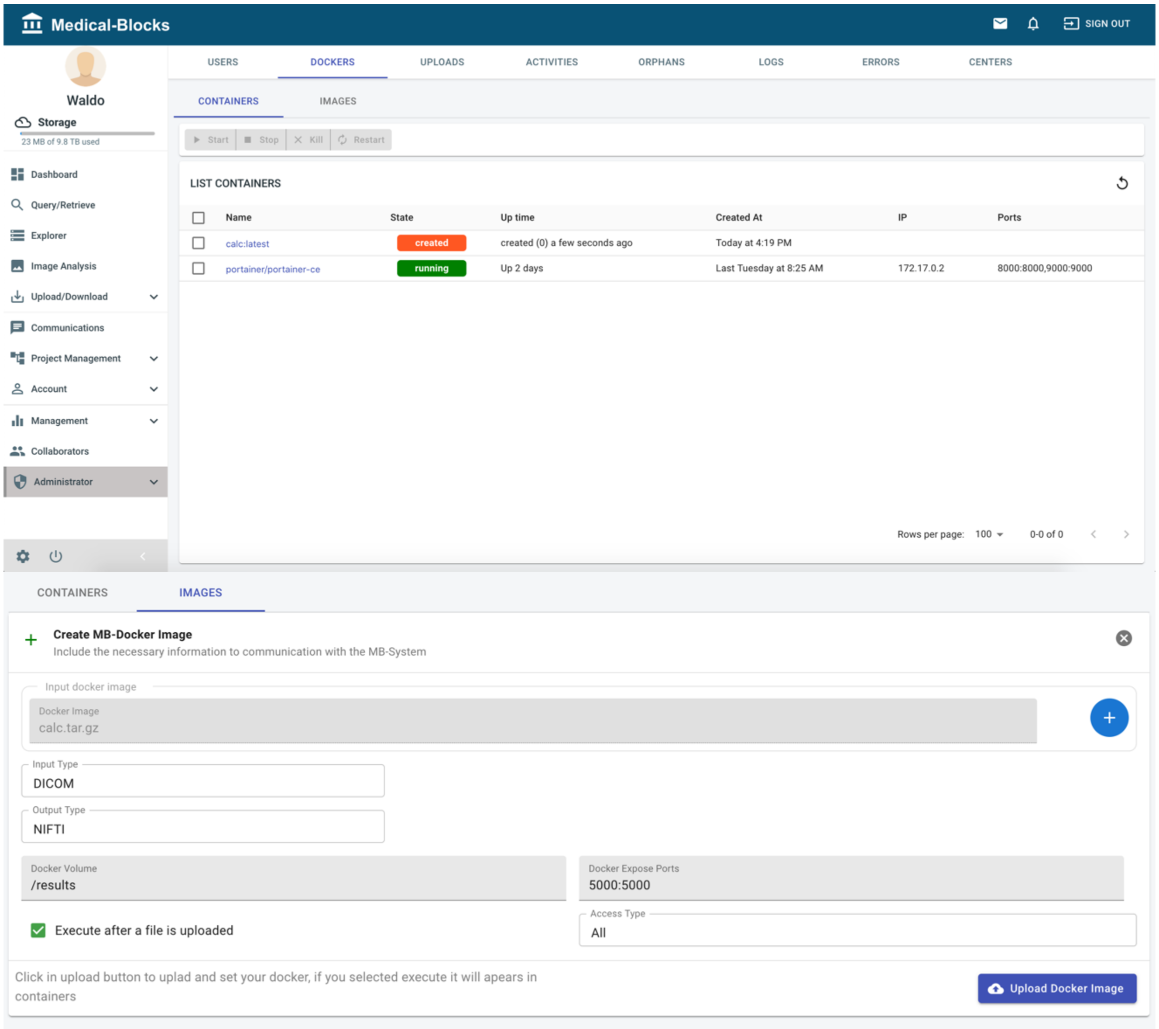

Figure 4: Managing of the Docker containers – the so-called blocks – inside the web UI. (top) Docker containers can be started, stopped, killed, and restarted. (bottom) Upload UI for new blocks. The user adds the Docker image and makes some basic configuration.

S3 Analysis blocks

The classification of data is performed automatically by blocks that extract the necessary metadata for the dashboard. The blocks are Docker containers and need to be programmed and integrated into Medical-Blocks as described in A2. One of the built-in analysis blocks is an imaging sequence detection, which extracts the DICOM tags SequenceName and SeriesDescription from the DICOM files, performs a mapping, and stores the "cleaned" sequence name in the internal database. The mapping is necessary as sequence names vary considerably between hospitals and can be edited in the web UI as shown in Figure 5. The block itself is a small C++ program using the Qt [9] and DCMTK [1] libraries to load the DICOM images and extract the DICOM tags in a JSON string, which is then mapped to a JavaScript object by the JSON.parse method. The mapping is stored in a SQL database, which the block accesses to return the mapped sequence name.

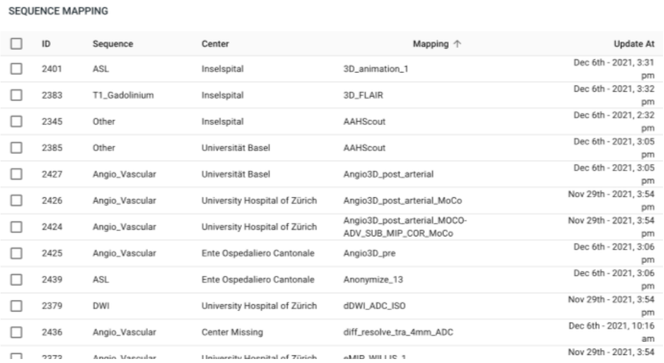

SEQUENCE MAPPING

ID	Sequence	Center	Mapping	Update At
2401	ASL	Inselspital	3D_animation_1	Dec 6th - 2021, 3:31 pm
2383	T1_Gadolinium	Inselspital	3D_FLAIR	Dec 6th - 2021, 3:32 pm
2345	Other	Inselspital	AAHScout	Dec 6th - 2021, 2:32 pm
2385	Other	Universität Basel	AAHScout	Dec 6th - 2021, 3:05 pm
2427	Angio_Vascular	Universität Basel	Angio3D_post_arterial	Dec 6th - 2021, 3:05 pm
2426	Angio_Vascular	University Hospital of Zürich	Angio3D_post_arterial_MoCo	Nov 29th - 2021, 3:54 pm
2424	Angio_Vascular	University Hospital of Zürich	Angio3D_post_arterial_MOCO-ADV_SUB_MIP_COR_MoCo	Nov 29th - 2021, 3:54 pm
2425	Angio_Vascular	Ente Ospedaliero Cantonale	Angio3D_pre	Dec 6th - 2021, 3:06 pm
2439	ASL	Ente Ospedaliero Cantonale	Anonymize_13	Dec 6th - 2021, 3:06 pm
2379	DWI	University Hospital of Zürich	dDWI_ADC_ISO	Nov 29th - 2021, 3:54 pm
2436	Angio_Vascular	Center Missing	diff_resolve_tra_4mm_ADC	Dec 6th - 2021, 10:16 am
[illegible]	[illegible]	[illegible]	[illegible]	Nov 29th - 2021, 3:54

Figure 5: The mapping of the sequence detection block can be edited in the UI.

S4 GraphQL playground

The GraphQL [10] playground can be used to access SQL data that is stored in Medical-Blocks. Using the interface provided by the Apollo server [4], data can be retrieved in the JSON format using the different resolvers of GraphQL. Figure 6 shows a query to retrieve image series from the SQL database. We remark that the playground is intended for technically well-versed personnel but allows very flexible extraction of data for research.

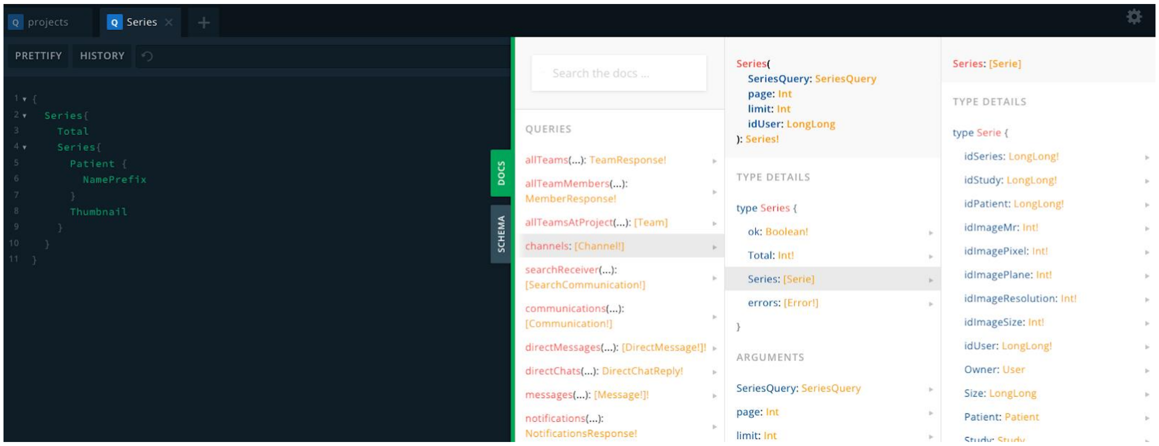

Figure 6: The GraphQL playground can be used to query the database of Medical-Blocks.

S5 MB-Sync and MB-SyncLight applications

MB-Sync consists of five Qt-based [9] libraries: sync, query, subscription, upload/download, and anonymization. The sync library, based on QFileSystemModel, sends an automatic message to the upload library anytime a file is modified in the sync folder of MB-Sync on the file system. If the data need to be anonymized, the anonymization library is called before the data is compressed and sent to Medical-Blocks through the upload/download library. The subscription library is used for real-time communication between Medical-Blocks and MB-Sync. Every time that a new file arrives to Medical-Blocks, MB-Sync instances are notified, and data is automatically downloaded to the sync folder if necessary. The HTTPS communication it is based on the QNetworkAccessManager library of Qt.

The main difference between MB-Sync and MB-SyncLight is that MB-SyncLight can only retrieve data but not upload or modify data. Therefore, the anonymization and

upload libraries are not present in MB-SyncLight, and the query library is only used to validate the shared link with the Medical-Blocks.

S6 MB-Connect application

The MB-Connect plugin is integrated into an in-house DICOM viewer called MB-Viewer (Figure 7) to import data into the cloud instance of Medical-Blocks to comply with the legal regulations when medical data is leaving the hospital IT network. Basically, MB-Viewer is a DICOM viewer with a connection to the PACS, extended by anonymization and import functionalities provided by MB-Connect. As MB-Viewer is a DICOM node, any PACS can send data to it. Therefore, by using MB-Viewer, patients can be queried and retrieved from the PACS at the Inselspital, Bern University Hospital. The query/retrieve dialog is equal to the dialog implemented in Medical-Blocks (cf. Figure 6 in the main text). It is further possible to use a batch functionality, which allows to query and retrieve multiple patients for cohort analysis. MB-Viewer does not require a login to the PACS, the application is simply installed on a computer in the hospital's IT network, the DICOM node is registered in the PACS, and MB-Viewer can then be used to query and view medical images. However, to import data into Medical-Blocks by MB-Connect, login credentials of Medical-Blocks are required. MB-Connect connects then to the back-end API of Medical-Blocks and sends data to it. MB-Viewer is a multi-platform application implemented in C++ that runs on Windows, macOS, and Linux operating systems. The same libraries for the PACS connection and the handling of DICOM image files as presented in A1 are used.

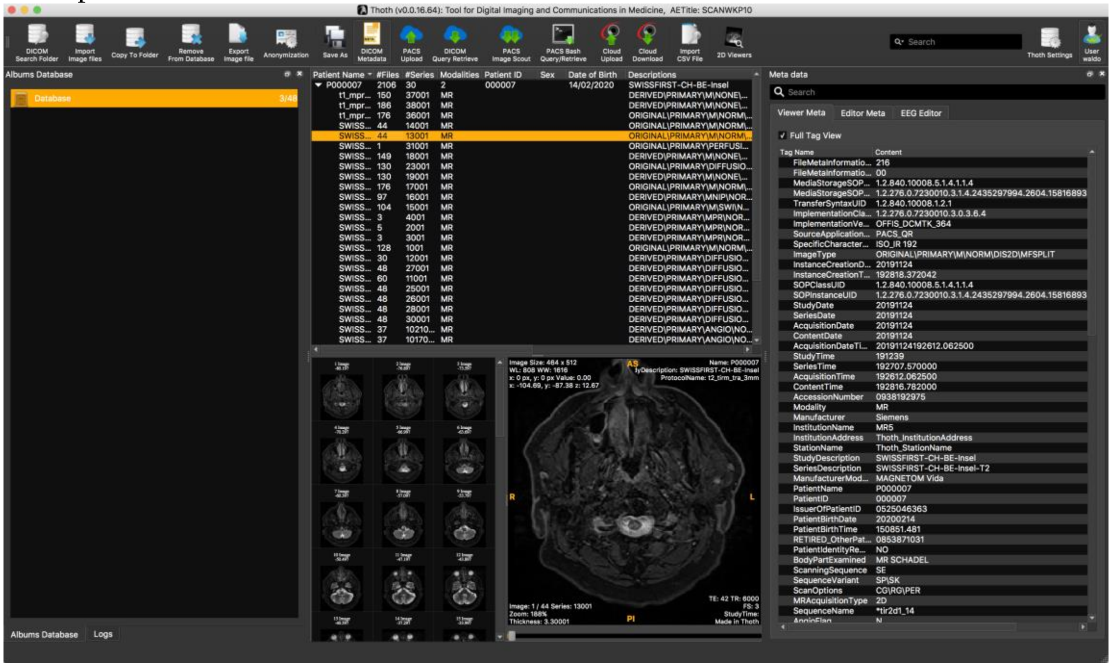

Figure 7: The MB-Viewer DICOM viewer with PACS exploration. The PACS can be queried, medical images, and their image properties can be viewed with MB-Viewer.

S7 Access rights and secure login

The access rights in Medical-Blocks are designed around the user, meaning that each file is associated to the user that uploaded the file. This user is the owner and administrator of the data. Like operating systems, user rights can be given by the owner to other users at

the level of read, write, delete, and download. It is also possible to provide access rights to files at the level of projects and teams. Technically in the SQL database, each file has a user ID associated and there exist joint tables that relate project IDs to file IDs as well as team IDs to file IDs.

To use the Medical-Blocks web UI, MB-Sync or the GraphQL playground provided by Apollo, a secure login system was created to access the different endpoints of the back-end (GraphQL and REST APIs). Furthermore, the Apollo server provides a secure second security layer to access different resolvers, by using the SchemaDirectiveVisitor that controls the access to the different resolvers and variables based on the user administration level. After a successful login, the user receives an encrypted authorization token and a refresh token that must be included in the HTTPS header every time the user makes a request to the back-end. The token login system bases on the JSON Web Token (JWT) library [11].

References

1. DCMTK - DICOM Toolkit [Internet]. [cited 2022 Jan 25]. Available from: https://dicom.offis.de/dcmtk
2. dcm4che-dockerfiles/dcm4chee-arc-psql [Internet]. [cited 2022 Jan 20]. Available from: https://github.com/dcm4che-dockerfiles/dcm4chee-arc-psql
3. Sequelize [Internet]. [cited 2022 Jan 20]. Available from: https://sequelize.org/
4. Apollo GraphQL [Internet]. [cited 2022 Jan 20]. Available from: https://www.apollographql.com/
5. European Data Format (EDF) [Internet]. [cited 2022 Jan 20]. Available from: https://edfplus.info/
6. Empowering App Development for Developers | Docker [Internet]. [cited 2021 Jul 13]. Available from: https://www.docker.com/
7. Container Management | Kubernetes GUI | Docker Swarm GUI | Portainer [Internet]. [cited 2022 Jan 25]. Available from: https://www.portainer.io/
8. Node.js [Internet]. [cited 2022 Jan 25]. Available from: https://nodejs.org/en/
9. Qt | Cross-platform software development for embedded & desktop [Internet]. [cited 2022 Jan 20]. Available from: https://www.qt.io/
10. GraphQL | A query language for your API [Internet]. [cited 2021 Jul 13]. Available from: https://graphql.org/
11. JSON Web Tokens [Internet]. [cited 2022 Jan 20]. Available from: https://jwt.io/
